# Taxes on Sugar-Sweetened Beverages to Reduce Overweight and Obesity in Middle-Income Countries: A Systematic Review

**DOI:** 10.1371/journal.pone.0163358

**Published:** 2016-09-26

**Authors:** Sharon S. Nakhimovsky, Andrea B. Feigl, Carlos Avila, Gael O’Sullivan, Elizabeth Macgregor-Skinner, Mark Spranca

**Affiliations:** 1 International Health Division, Abt Associates, Bethesda, Maryland, United States of America; 2 Department of Global Health and Social Medicine, Harvard Medical School, Boston, Massachusetts, United States of America; 3 Office of Reputational Capital, Abt Associates, Cambridge, Massachusetts, United States of America; Centro de Investigacion y de Estudios Avanzados Unidad Merida, MEXICO

## Abstract

**Background:**

The consumption of sugar-sweetened beverages (SSBs), which can lead to weight gain, is rising in middle-income countries (MICs). Taxing SSBs may help address this challenge. Systematic reviews focused on high-income countries indicate that taxing SSBs may reduce SSB consumption. Responsiveness to price changes may differ in MICs, where governments are considering the tax. To help inform their policy decisions, this review compiles evidence from MICs, assessing post-tax price increases (objective 1), changes in demand for SSBs and other products, overall and by socio-economic groups (objective 2), and effects on overweight and obesity prevalence (objective 3).

**Methods and Findings:**

We conducted a systematic review on the effectiveness of SSB taxation in MICs (1990–2016) and identified nine studies from Brazil, Ecuador, India, Mexico, Peru, and South Africa. Estimates for own-price elasticity ranged from -0.6 to -1.2, and decreases in SSB consumption ranged from 5 to 39 kilojoules per person per day given a 10% increase in SSB prices. The review found that milk is a likely substitute, and foods prepared away from home, snacks, and candy are likely complements to SSBs. A quasi-experimental study and two modeling studies also found a negative relationship between SSB prices and obesity outcomes after accounting for substitution effects. Estimates are consistent despite variation in baseline obesity prevalence and per person per day consumption of SSBs across countries studied.

**Conclusions:**

The review indicates that taxing SSBs will increase the prices of SSBs, especially sugary soda, in markets with few producers. Taxing SSBs will also reduce net energy intake by enough to prevent further growth in obesity prevalence, but not to reduce population weight permanently. Additional research using better survey data and stronger study designs is needed to ascertain the long-term effectiveness of an SSB tax on obesity prevalence in MICs.

## Introduction

Consumption of sugar-sweetened beverages (SSBs) is rising in middle-income countries (MICs) [1]. Drinking SSBs can lead to weight gain and increase the risk of type-2 diabetes and cardiovascular disease [2, 3]. A modeling study attributed 184,000 annual deaths worldwide to SSB consumption in 2010, mostly due to type-2 diabetes (72%) or cardiovascular disease (24%); 71% of these deaths occurred in MICs [4]. SSB consumption may rise further in MICs where multinational companies are targeting new investments [5], potentially accelerating the increase in the morbidity and mortality from obesity, type-2 diabetes, and cardiovascular disease. At the same time, other drivers of obesity–including economic growth and urbanization–continue to change food and workplace environments in MICs, exacerbating the imbalance between higher food intake and lower levels of physical activity [6].

Taxing SSBs to improve health outcomes at the population level has emerged as a cost-effective intervention to address the rising prevalence of obesity [7]. The World Health Organization (WHO) promotes taxes as among the most effective means of controlling tobacco and alcohol use [8, 9]. Recently, the WHO and others also called for taxing SSBs, arguing that the intervention may generate similar value for public health [10–13]. SSBs are a sensible target for taxation because they are calorically dense and have no additional nutritional value [3, 12]. Also, people generally do not reduce their consumption of other calories after drinking SSBs, thus increasing the amount of excess energy consumed [14]. MIC governments of Nauru, Mexico, and Dominica have already implemented taxes on SSBs [15]. Additionally, governments of South Africa, Thailand, and Vietnam are actively considering them [16–18].

**Fig 1:**
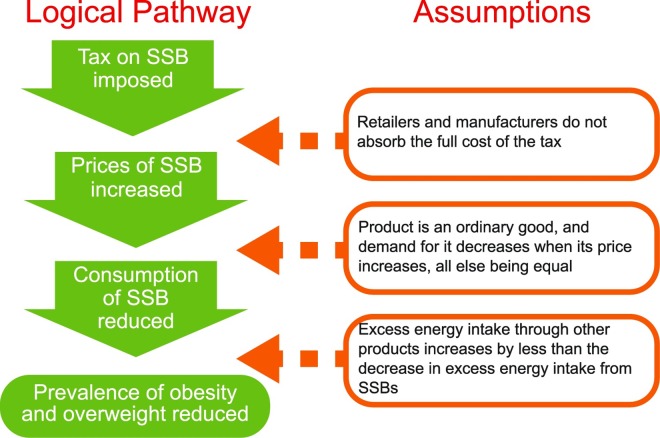
Logical Pathway from Taxing SSBs to Public Health Impact.

The logical pathway in Fig 1 illustrates how taxes on SSBs can reduce the prevalence of obesity and overweight if several assumptions hold. The pathway is specific to excise taxes which, unlike sales or value added taxes, increase SSB prices relative to other goods [19] and are recommended by the WHO for tobacco control [20]. The pathway first requires that the tax is not fully absorbed by producers and will thus lead to an increase in consumer prices. Given imperfect market competition, this theory postulates that excise taxes can lead to price increases as large or larger than the tax rate when the taxed good, like SSBs, contains many similar, but not identical, products [21]. Still, the extent to which prices increase relative to the tax rate (the “pass-through rate”) depends on factors such as consumers’ responsiveness to price change, which may vary in different socio-economic settings, and the costs producers face [22].

Secondly, the pathway assumes that SSBs are ordinary goods: the higher the price, the lower the demand. To determine the direction and magnitude of consumers’ response to SSB price increases, economists estimate own-price elasticity (PE), which is the percentage change in the consumption of SSBs over percentage change in SSB prices.

Finally, the pathway requires that the tax leads to a significant net reduction in excess energy intake, the difference between calories consumed and burned, even after “substitutes” and “complements” are taken into account. Substitutes are products whose consumption rises when SSB prices increase and consumers shift away from the higher priced good to alternatives, such as milk, unsweetened juice, and bottled water. Complements are products such as fast food whose consumption rises and falls with SSB products. Substitutes reduce, and complements increase, the extent to which net consumption of excess energy falls given increases in SSB prices. Cross-PE, defined here as the percentage change in the consumption of another food or beverage over percentage change in SSB prices, indicates the relationship between other goods and SSB prices. A good with a negative cross-PE with SSBs is considered a substitute to SSBs, since its consumption decreases as SSB prices increase. A good with a positive cross-PE with SSBs is considered a complement to SSBs, since its consumption increases as SSB prices increase.

Existing systematic reviews, primarily based on studies from high-income countries, indicate that taxes on SSBs may reduce SSB consumption. However, these reviews show less conclusive results related to the consumption of substitutes and complements and the downstream effects on obesity outcomes [23–26]. While some studies argue that the tax may work similarly in MICs and high-income countries [25], other studies have predicted that the tax may be more effective in MICs, given the evidence that consumers are more responsive to prices changes in lower-income countries [27–29]. Another study argues that baseline tax rates and levels of SSB consumption and obesity are key variables that will shape the degree to which the tax is effective in reducing obesity—variables which vary widely across MICs [16].

This systematic review aimed to synthesize existing evidence on the effectiveness of an SSB tax in MICs, in comparison to the evidence from high-income countries. Specific objectives were to assess whether, in MICs 1) prices increase after governments impose an excise tax; 2) net intake of excess energy falls across the population, and whether the magnitude of change differs across socio-economic groups; and 3) obesity outcomes fall as a result of increasing SSB prices. This review contributes to existing literature by achieving these objectives and by including several recently conducted studies from MICs that update other systematic reviews [30].

## Methods

This review followed the guidelines for the Preferred Reporting Items for Systematic Reviews and Meta-Analyses (PRISMA) [31]. It updated a 2013 systematic review by Maniadakis et al., adapting the research protocol to MICs and to taxes on SSBs, excluding those targeting unhealthy foods [30]. No separate research protocol for this review was published. This review does not include a meta-analysis due to variation in the targeted products, methods for estimating consumption, study designs, and difficulties in controlling for income, population, and other country-specific characteristics. The PRISMA checklist appears in S1 Table.

### Selection of Studies

This review included studies from MICs that measured the association of taxes on or prices of SSBs with their consumption and, where possible, the outcomes reflecting percentage change in body mass index (BMI), prevalence of obesity, or prevalence of obesity and overweight [32]. Countries were classified as MICs according to World Bank definitions [32]. BMI is a measure of weight (in kilograms) over height (in meters squared) that is used to define overweight (BMI ≥ 25) and obesity (BMI ≥ 30). Independent variables included either market or policy-driven price changes; policy-driven price changes may be taxes whose primary purpose is to raise revenue or promote health. This review also assessed the relationship between tax and price, an intermediate outcome in the logical pathway to health impact. This review included studies that focus on “SSB products” defined as an overlapping group of beverages such as “soda,” referring to all carbonated soft drinks, sugary and diet, and “sugary soda,” referring only to carbonated soft drinks with added sugar. This review only included studies based on primary, quantitative research written in English. All such studies—modeling, non-experimental, quasi-experimental, or experimental studies, and published journal articles, dissertations, or working papers—meeting these criteria were included. This review excluded other systematic reviews and meta-analyses as well as qualitative studies, case studies and reports, and letters to the editor.

### Search Strategy and Data Sources

The search for studies meeting the criteria for inclusion first identified studies from MICs about SSBs that were included in a global systematic review covering the period January 1990 to February 2013 [30]. The search protocol from this review was adapted to include studies from MICs and about SSBs only, and was applied to March 2013 and March 2016. Databases included PubMed, Web of Science, Cochrane Library, AgEcon, EconLit, the National Agricultural Library, and Google Scholar. Finally, the reference lists in the selected studies were checked and leading scholars contacted to identify any additional studies. One author (S.S.N.) conducted the search, importing all identified titles and abstracts into EndNote, removing duplicates, and screening all titles and abstracts. S. S.N. then reviewed the full text of all the remaining articles, excluding those that did not meet the eligibility criteria for this review. Another author (A.B.F.) provided guidance on selection decisions and independently reviewed 18 full text articles, of which nine were selected as the final sample. The search algorithm was adapted from the one used in Maniadakis (2013) [33]. It requires that the title or abstract in the studies include a relevant financial, nutritional, and outcome term. Table 1 lists the pseudocode for the search algorithm used in this review.

**Table 1:** Search Algorithm.

**Term 1 (Financial)**	Tax OR taxes OR taxing OR taxation OR price OR prices OR pricing OR economic OR financial OR fiscal OR penalty OR penalties
**AND**	
**Term 2 (Nutritional)**	Sugar OR sugar OR sweetened OR carbonated OR soft OR sucrose OR soda OR sodas OR cola OR colas OR drink OR drinks OR beverage OR beverages
**AND**	
**Term 3 (Outcome)**	Intake OR consumption OR demand OR quantity OR quantities OR sale OR sales OR habit OR habits OR behavior OR diet OR nutrition OR calorie OR calories OR elasticity OR elasticities OR weight OR overweight OR obese OR obesity OR body mass index

### Data Extraction and Synthesis

Two authors (S.S.N. and A.B.F.) extracted the following key information from included studies: i) study background: author, year of publication or draft, country, objectives, and funding source; ii) a description of the data: population, sample size, and type and year of data; iii) study design: quasi-experimental with before-and-after effects measured using statistical methods even if there was no control group, non-experimental where the statistical methods were not based in before-and-after or control-treatment comparisons, and modeling studies which applied assumptions to baseline cross-sectional data; iv) definitions of independent and key outcome variables, including tax pass-through rate, own-PE and cross-PEs, and change in BMI and overweight and obesity prevalence; v) estimates of key outcomes for the whole population and for socio-economic groups if possible; and vi) study conclusions.

To make objective one results more policy relevant, authors standardized estimates for change in the consumption of SSB products in kilojoules per person per day (kJ PPPD) given a 10% change in SSB prices. Two quasi-experimental studies [34, 35] and one non-experimental study [36] reported change in consumption given a 10% change in SSB product prices in volume (liters or milliliters) or kilocalories. For these studies, standardization required making simple conversions: i) from liters to kilocalories based on nutritional information from a soft drink and beverage product company (1 milliliter of product has 0.4 kilocalories) [37]; ii) from kilocalories to kJ (1 kJ is equivalent to .24 kilocalories) [38]; iii) from per person per month to per person per day (1 month has an average of 30.4 days); and iv) from per household per day to per person per day based on average size of the household reported in the study. One modeling study [17] reported change in consumption given a 10% change in SSB prices in kJ; authors assumed a linear relationship between percentage change in price and percentage change in consumption, dividing this estimate in half. Authors made the same assumption of linearity between percentage change in price and consumption when standardizing estimations from a modeling and non-experimental study [39, 40]. For these studies, authors calculated the change in kJ PPPD given a 10% change in SSB prices by multiplying own-PE estimates with baseline consumption of SSBs PPPD, and then converting using the same conversions above. Three studies did not include sufficient information to allow for standardization [41, 42]. This additional analysis was not pre-specified.

### Assessment of Study Quality

Study quality was primarily determined based on study design, with the quasi-experimental studies ranked first and highest and the non-experimental studies and modeling studies ranked second and lowest. Study quality was also determined using the quality checklist for food and beverage taxes and subsidies studies in a previous systematic review [23]. The quality checklist was adapted to differentiate among the large number of studies based on cross-sectional data by reporting the number of time points each study estimated within its study period. In addition, authors assessed the quality of the statistical methods used among all study types.

### Ethics Statement

This review did not require an ethics review as it exclusively used published secondary materials.

## Results

**Fig 2:**
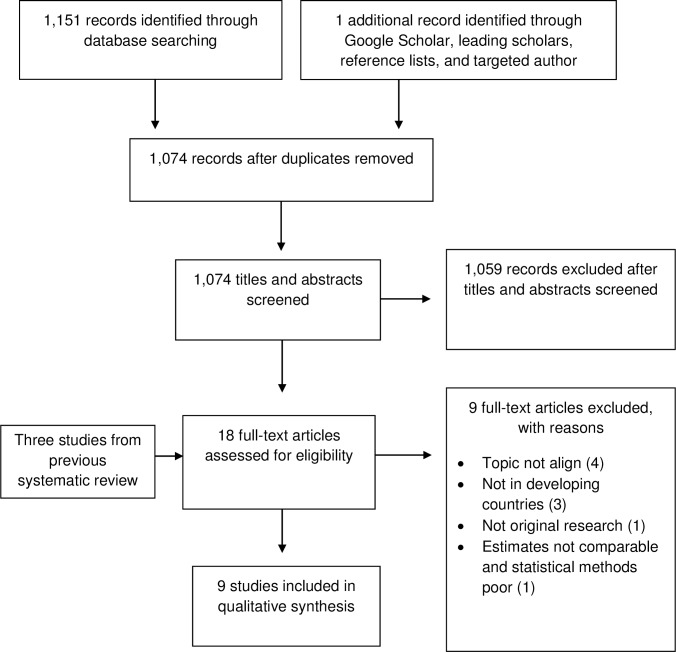
Documentation of Study Search and Selection.

Database searches for relevant studies published between March 2013 and March 2016 initially identified 1,151 records (Fig 2). Another study was included from a reference list. Seventy-eight duplicates were removed and, based on a review of titles and abstracts, 1,059 records were excluded because they did not align with this review or data were not from MICs. The full text of the remaining 15 records, along with the three studies focused on MICs from the previous systematic review (period 1990–2013), was then reviewed. Three of these 18 studies were excluded because they were not from MICs, six were excluded because scopes did not align, and one was excluded because the study type did not match the inclusion criteria. Additionally, one study was excluded because its statistical methods were weak. Specifically, this study did not estimate the relationship between price and consumption of soda directly. Rather, it estimated average changes in the prices and consumption of soda relative to other goods over an eight year period (1992–2000). This study also did not report statistical significance, nor did it define the SSB product studied in detail [43].

### Characteristics of Included Studies

Characteristics of the nine studies included in this review are presented in Table 2. One study measures the relationship between tax and price [41]. All studies measure the relationship between tax or price and the consumption of targeted products, and three studies measure the relationship between tax or price and outcomes related to obesity [17, 34, 40]. Five studies focus on SSBs as a group [17, 35, 39, 40, 44] and two studies focus on sugary soda [36, 41]. Due to data constraints, one study focuses on soda [34] and one study considers SSBs together with diet soda [45].

**Table 2:** Tabular Summary of Studies Included in the Systematic Review.

• Authors, Year • Publication Type	Objectives	Data on SSB consumption and study population	1. Country 2. Methods 3. Food and beverage products studied	Independent and outcome variable			Consumption (and health status/risk factor) related outcome	Conclusions
**1. Quasi-experimental, observational studies**								
• Colchero et al, 2016 • Published paper	Estimate changes in the purchasing of taxed and untaxed beverages since implementation of Mexico's 2014 1 peso per liter specific excise tax (~9/10%) on nondairy SSBs	• Commercial, unbalanced panel data with a sample of 6,253 households from 53 cities, representing 63% of Mexico's population and providing 205,112 household-month observations • Sub-population: socio-economic status thirds	1. Mexico 2. Before-and-after longitudinal study 3. Carbonated and non-carbonated SSBs; and some non-taxed beverages (e.g., bottled water)		• Prices of purchased products • Volume (mL) per capita per day of beverages purchased, logged	• Compared to the counterfactual, purchases of taxed beverages were 6.1% (12 mL per capita per day (PPPD)**) lower across the first year; this difference accelerated to 12% (22 mL PPPD**) in December 2014. Purchase reduction was higher in the lowest socio-economic status third (average 9% (19 mL PPPD)* lower; 17% (35 mL PPPD)* lower in December). • Purchases of non-taxed beverages increased but not significantly from zero.		Purchases of taxed items decreased in the short term (1 year) at an increasing rate. Further monitoring of the tax's effect over a longer period, including changes in packaging and pricing by manufacturers and retailers, is needed.
• Grogger, 2015 • Published working paper	Observe how prices of soda and other beverages responded to Mexico's 2014 1 peso per liter specific excise tax (~9/10%) on nondairy SSB to determine whether the tax was passed on to consumers and to understand the tax's effect on the consumption of beverages	• Mexico's Consumer Price Index with retail prices from 46 cities in Mexico • Sub-population: none	1. Mexico 2. Before-and-after study of prices 3. Taxed products: soda and other taxed drinks, diet drinks, bottled water, and milk		• Time (before and after tax implementation began) • Real prices relative to December 2013	• After the first 15 months of the tax, prices of soda increased by 12% (1.32* and 1.42* peso increase by 2014 and 2015, respectively, relative to December 2013 prices). Prices of other drinks with added sugar increased significantly in 2014, but insignificantly in 2015. • Increases in the prices of diet soda (0.61* and 0.64* peso increase by 2014 and 2015, respectively) indicate potential substitution. Milk prices increased significantly in 2014 only, while juice prices increased neither year.		Results show the tax on soda was over-shifted to consumers, indicating that the tax likely had a "relatively powerful effect." There was little evidence of substitution to other caloric beverages, which suggests a drop in consumption of soda was not balanced by increased consumption of other drinks and the tax may in fact reduce total caloric consumption.
• Ritter Burga, 2016 • Published dissertation	"Determine whether a reduction in the price of carbonated soft drinks (CSDs) increases obesity rates, through the increase in CSD consumption"	• Households whose women (female head or wife of head) are between ages of 19 to 49 years and were sampled in the nationally representative 1997 and 2001 National Household Survey (ENAHO) of Peru (n = 19,658) • Sub-population: households with and without access to water	1. Peru 2. Before-and-after repeated cross-sectional study 3. CSD (excluding soda water, including diet soda)		• Six-month average real index price of CSD • CSD liters per capita per month; BMI and obesity prevalence; diarrhea prevalence	• The 10% drop in six-month average prices of CSD 1997–2001 led to a 90 mL per person per month (PPPM)* increase in CSD consumption across all households (n = 28,000) and 120 mL PPPM* for households with women aged 19–49. The price drop also led to an 8.5%† increase in obesity prevalence and 14.8%† increase in BMI. • The drop in price shows food prepared away from home to be complementary with CSD (drop by 0.07 kg PPPM*). No effect was detected for alcohol, milk, or the combined category of bottled water and juice.		Results demonstrate "a causal relationship between the decrease in CSD price and the increase in obesity prevalence" through increased CSD consumption. They also indicate that the population is responsive to changes in price of CSD, without risk of substitution to milk and alcohol, making taxes a promising policy option to reduce obesity. However, a price increase in CSD may also increase diarrhea prevalence among households without access to piped water, indicating the policy may not avoid troubling side effects for health equity.
**2. Non-experimental, observational studies and modeling studies**								
• Barquera et al., 2008 • Published article	Increase understanding of patterns in consumption of beverages, including but not limited to SSBs, by Mexican adolescents and adults	• Three nationally representative Mexican HouseholdIncome and Expenditure Surveys conducted in 1989 (n = 11,501), 1998 (n = 10,919), and 2006 (n = 20,349). • Sub-populations: socio-economic thirds	1. Mexico 2. Repeated cross-sectional study 3. Soda, sweet drinks, whole milk, juice, bottled water		• Prices as reported in survey data • Expenditure on soda, to estimate own- and cross-PE for soda and whole milk with other beverages		• Own-PE increased over time, from -0.61* in 1989, to -0.85*in 1998 and -1.085* in 2006. The 2006 elasticity translates to an average 50 mL* drop per household per day in soda consumption given a 10% increase in price. Drop was slightly larger (53 mL*) for the lowest third of households compared to highest third (46 mL*). • Soda cross-PE was steady over the study period. Results indicate that sweet drinks (-0.11*) and juice (-0.02*) are complements while whole milk (0.52*) and bottled water (0.02) are substitutes (2006 estimates).	Own-PE of soda and whole milk are "modest but increasing," with soda more responsive to price change than whole milk. Cross-PEs of soda indicate that increasing consumption of other caloric beverages would be smaller than the decrease in soda consumption. This indicates that a tax may contribute to reducing obesity.
• Basu et al, 2014 • Published article	Estimate potential long-term health effects of taxing SSB in India, including calculations of own- and cross-PE.	• The nationally representative Indian National Sample Survey for 2009–2010, with 100,855 households • Sub-population: urban and rural, men and women, age groups, income groups	1. India 2. Simulation based on cross-sectional data 3. SSBs		• Prices as reported in survey data • Own- and cross-PE for SSBs; type 2 diabetes prevalence; type 2 diabetes incidence; impact on overweight and obesity prevalence, and type-2 diabetes incidence		• SSB own-PE was -0.94*; SSB cross-PE with fresh fruit juice was 0.31*, with milk 0.049*, and with tea: 0.13*. • A conservative estimate of impact of a 20% SSB tax between 2014–2023 is a 3% (95% CI 1.6–5.9%) reduction in overweight and obesity prevalence; and a 1.6% (95% CI1.2–1.9%) reduction in diabetes incidence, with largest impact among young rural men	Taxing SSBs is a promising intervention. In relation to other interventions, this tax may have a more sustained impact on overweight, obesity, and type 2 diabetes incidence in India, with health benefits reaching rural and poor populations.
• Claro et al., 2012 • Published article	Quantify the effect of prices on consumption of SSBs in Brazil to contribute to discussion on applicability of SSB tax in middle income countries	• Individual and economic data from the Households Budget Survey 2002–2003, with 443 strata of households, composed of 48,470 households that were "geographically and socioeconomically homogeneous" • Sub-population: households by income quartiles	1. Brazil 2. Cross-sectional study 3. SSBs		• Mean prices of SSBs (reals) per 1,000 kcals); and mean income (reals per person per month) • Kcals per adult equivalent per day, to estimate own-price and income elasticities for SSB		• Results show that SSB own-PE is -0.85* for the full population. It is -1.03* for the poorest quintile and -0.63 for all other quintiles. • SSB income elasticity was 0.41*, indicating that rising income drives increases in SSB consumption.	Taxing SSBs is a promising intervention in Brazil; results indicate it could contribute to reducing consumption of SSBs, especially for poorer populations.
• Colchero et al., 2015 • Published article	Estimate own- and cross-PE of demand for SSB in Mexico between 2006 and 2010	• The 2006, 2008, and 2010 Mexican National Income and Household Expenditure Surveys with 19,512, 27,994, and 25,805 households in the samples, respectively. • Sub-population: urban/rural, income, marginality index	1. Mexico 2. Repeated cross-sectional study 3. Soft drinks (sugar-sweetened and diet), other SSBs, water, milk, candies, snacks, sugar, and traditional snacks.		• Prices as reported in survey data • Expenditure on SSBs, to estimate own-PE and cross-PE for sodas and SSBs		• Across study period, SSB own-PE was -1.16**. Estimates increased as the population became more marginalized (own-PE in second most marginalized group was -1.41**). • SSB cross-PE were positive for milk (0.19**) and natural and mineral water (0.1**), indicating some substitution; they were negative for all food products (e.g., candies (-0.44**) and snacks (0.23**)), indicating complementarity.	"Given high rates of consumption of SSB/soft drinks and of overweight and obesity, a tax could reduce consumption and have a positive effect on health."
• Manyema et al, 2014 • Published article	"Estimate the effect of a 20% tax on SSBs on the prevalence of and obesity among adults in South Africa"	• Individual data from the 2012 South African Health and Nutrition Examination Survey, which is based on data from 25,532 individual respondents • Sub-population: gender, age	1. South Africa 2. Simulation based on cross-sectional data and meta-analysis results 3. SSBs		• 20% tax on SSB (with 90%, 100%, and 110% pass-through rate variations) • kJ PPPD; and BMI		"A 20% tax is predicted to reduce energy intake by about 36 kJ per day (95% CI 9–38 kJ). Obesity is projected to reduce by 3.8% (95% CI 0.6–7.1%) in men and 2.4% (95% CI: 0.4–4.4%) in women."	Taxing has promise as an effective intervention, in the context of a "multi-faceted effort to prevent obesity"
• Paraje, 2016 • Published article	Estimate own-PEs for SSBs in Ecuador to predict effect of a tax on SSBs	• Sample of 39,434 households from the 2011–2012 National Urban and Rural Household Income and Expenditures Survey • Sub-population: lowest 40%, middle 40% and highest 20% income groups	1. Ecuador 2. Cross-sectional study 3. SSBs, specific SSB products including energy drinks, colas and carbonated beverages, and concentrated fruit juices		• Expenditure per liter (proxy for SSB prices) • Average quantity (in liters) of SSBs		• Own-PE was -1.2** for full population, -1.3** for the lowest income group, -1.2*** for the middle income group, and -1.2 for the top income group. • SSB income elasticity is estimated at 0.79**, indicating that rising income drives increases in SSB consumption.	Estimates suggest that taxing SSBs in Ecuador could reduce consumption of SSBs and contribute to public health objectives.

*Significantly different from zero at the 5% level.

** significantly different from zero at the 1% level

† significantly different from zero at the 90% level.

The studies reviewed investigated SSB prices and consumption in Brazil (n = 1) [39], Ecuador [44], India (n = 1) [40], Mexico (n = 5) [35, 36, 41, 43, 45], Peru (n = 1) [34], and South Africa (n = 1) [17]. In 2014, consumption of SSBs ranged from very high in Mexico, Brazil, and Ecuador (661, 337, and 324 kJ PPPD, respectively) to very low in India (21 kJ PPPD) [1, 44]. The countries represented in this review also vary in baseline overweight (22–64.5%) and obesity prevalence (5%-28%), with India measuring lowest and Mexico measuring highest for both indicators [46].

### Quality of Included Studies

This review first ranked the quality of the studies based on study design (Table 3). All included studies were observational or modeling studies, which limits their ability to causally attribute changes in consumption or obesity outcomes to the policy or market-driven price changes observed. Within this review, three quasi-experimental studies have the highest quality, but employed different methodologies. Two of these studies measure the before-and-after effects of Mexico’s 1 peso per liter 2014 excise tax on SSBs. One used panel data of household consumption [35] and the other used price data to observe tax incidence and identify indirect evidence of changing consumption patterns [41]. The third quasi-experimental study leveraged an exogenous event causing a drop in prices to measure the before-and-after effects across treatment and comparison groups and draw causal inferences [34]. One of these studies is a published working paper [41] and another is a published dissertation [34]. In addition, this review included four non-experimental and two modeling studies that rely on variation in price and consumption within and between specific beverages and demographic or income groups to estimate consumption outcomes [17, 36, 39, 40, 44, 45].

**Table 3:** Quality Assessment of Studies.

Study Type (1 highest quality, 2 lowest quality)	Quasi-experimental, observational studies (1)			Non-experimental, observational studies and modeling studies (2)					
Author, Year; Country	Colchero, 2016; Mexico	Grogger, 2015; Mexico	Ritter Burga, 2016; Peru	Barquera, 2008; Mexico	Basu, 2014; India	Claro, 2012; Brazil	Colchero, 2015; Mexico	Manyema, 2014; South Africa	Paraje, 2016; Ecuador
Is the study design prospective?	√	-	-	-	-	-	-	-	-
How many time points does the study include and over what time period?	36/36 (months)	51/51 (months)	2/5 (years)	3/18 (years)	1/1 (years)	1/1 (years)	3/5 (years)	1/1 (years)	1/1 (years)
Does the data include all SSBs consumed, or just a subset?	-	-	-	√	√	-	√	√	**-**
Do price and consumption data come from the same population?	√	√	√	√	√	√	√	-*	**√**
Does the study consider potential substitution to other products?	√	√**	√	√**	√**	-	√**	√**	**-**
Are the effects for each SSB product analyzed separately?	√	√	-	√	√	-	√	-	**√**
Does the study assess an actual tax?	√	√	-	-	-	-	-	-	-

*Uses meta-analysis for own- and cross-PE estimates

**study considers substitution to non-alcoholic beverages, but not to alcoholic beverages and/or food products

All but two studies [39, 44] considered substitution effects, albeit to varying degrees. All studies considered other non-alcoholic beverages, but only one considered substitution to alcoholic beverages, and only two considered substitution to food products. In comparison, a global systematic review found that only four of 16 studies on SSB taxation investigate the substitution effects of an SSB tax [23]. Three of the nine studies included all SSB consumption by individuals or households, while the others are restricted to home consumption.

The quality of statistical methods used in the studies was also assessed. The three studies estimating the relationship between price change and obesity outcomes have several important limitations. First, these studies, one quasi-experimental and two modeling, did not control for some potential confounders, such as changes in physical activity levels, which could account for some of the effect detected. The modeling studies assumed constant own- and cross-PEs over time [40] and across socio-economic groups [17]. Additionally, one study relied on meta-analysis own- and cross-PE estimates, as local data on price and consumption were unavailable [17].

### Objective 1: Measuring Industry Response and Changes in Prices

This review found differences in the effects of excise taxes on the prices of sodas compared to the prices of other SSBs. One quasi-experimental study measuring the before-and-after effects of Mexico’s January 2014 excise tax found that the price of sugary soda increased by 1.3 pesos per liter (95% CI: 1.2–1.4) on average in 2014, higher than the tax rate of 1 peso per liter. The price of sugary soda increased further, to 1.4 pesos per liter (95% CI: 1.3–1.6), during the first quarter of 2015. In contrast, the prices of other SSBs increased by 0.6 pesos per liter (95% CI: 0.2–1.1) on average in 2014, an increase less than the tax rate. Moreover, the average increase in the prices of other SSBs was no longer significantly different from zero over the first quarter of 2015 [41].

### Objective 2: Measuring Changes in the Consumption of SSBs and Related Products

**Fig 3:**
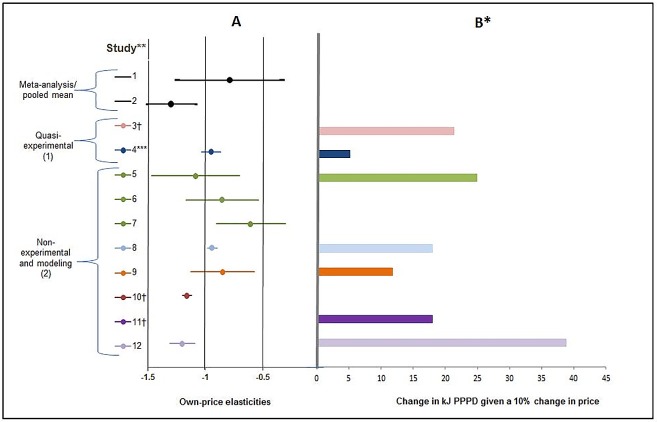
Own-Price Elasticities of SSBs and Change in kJ PPPD Given a 10% Price Increase. *Change in kJ PPPD given a 10% price change was calculated from study estimates using unit conversions based on the identities and assumptions presented in the methods section; **estimates come from the following studies listed as: author, (year of study), country in (year of estimate): 1 -Andreyeva et al. (2010) United States 1938–2007 [47]; 2—Escobar (2012) Global 2000–2013 [25]; 3—Colchero (2016) Mexico in 2015 [35]; 4—Ritter Burga (2016) Peru 1997–2001 [34]; 5—Barquera (2008) Mexico in 2006; 6—Barquera (2008) Mexico in 1998; 7—Barquera (2008) Mexico in 1989 [36]; 8 –Basu (2014) India 2014–2023 [40]; 9—Claro (2012) in 2003 [39]; 10—Colchero (2015) Mexico across 2006, 2008, and 2010 [42]; 11—Manyema (2014) South Africa in 2012 [17]; 12—Paraje (2016) Ecuador in 2012 [44]; ***findings based on observed increase in prices of soda; †missing estimates: Colchero (2016) results do not allow for a precise estimate of own-PE [35]; Barquera (2008) only provided analysis B estimates for 2006 data [36]; Colchero (2015) does not include baseline estimates of SSB consumption or other estimates needed for standardization [42]; Manyema (2014) [17] uses meta-analysis estimates for own-PE from Escobar (2012) [25], presented at the top of the figure; Grogger (2015) [41] was not included as its estimates do not include own-PE estimates and do not allow for standardization.

Consumption of SSBs Across the Population. Overall, these results showed a consistent negative correlation between price and SSB consumption. Fig 3 presents own-PE estimates for SSB products from six studies [34, 36, 39, 40, 44, 45]. Estimates range from -0.6 to -1.2. Two studies without own-PE estimates also demonstrated a negative relationship between the price and consumption of SSB products [35, 41]. Fig 3 also presents the change in kJ PPPD given a 10% price increase, with estimates ranging from 5 to 39 kJ PPPD and a median estimate of 18 kJ PPPD. Changes in SSB product consumption is higher (21–39 kJ PPPD) among studies from Mexico and Ecuador than in other countries (5–18 kJ PPPD) (Fig 3).

Consumption of SSBs by Socio-economic Group. Of the six studies with relevant estimates, a quasi-experimental study estimated that the reduction in SSB products purchased relative to a counterfactual is higher (9.1%) among the lowest socio-economic third than among the highest (5.5%) [35] (S1 Fig). Similarly, three non-experimental studies’ own-PE by quintile detected differences in sensitivity to price change by sub-population, with own-PEs generally higher among more vulnerable and lower income groups [39, 44, 45]. In contrast, a non-experimental study and a modeling study grouping socio-economic groups into thirds showed little difference in own-PE estimates across socio-economic groups [36, 40]. One of these studies noted under-sampling among the rural low-income group, which likely weakened its power to detect differences between sub-groups [40]. Overall, these findings suggest that lower socio-economic groups are more responsive to price changes in SSB products compared to other groups in MICs.

Changes in the Consumption of Other Products. Five studies in this review considered the relationship between milk consumption and SSB product prices (Table 4). A quasi-experimental study (Grogger 2015) found milk to be a complement; however, the estimate was only significantly different from zero for the first of two years after the tax was imposed [41]. Another quasi-experimental study (Ritter 2015) did not detect a relationship between milk and SSBs [34]. Two non-experimental studies and a modeling study estimated that milk was a substitute to SSB products using cross-PEs [36, 40, 45]. The absolute own-PE estimate was larger than the cross-PE estimate in each study. This suggests that any increase in milk consumption would be smaller than the decrease in SSB consumption, given an increase in SSB prices and no other changes affecting consumption patterns.

**Table 4:** Changes in Consumption of Other Foods and Beverages Given Changed Prices of SSBs.

Study Type*	• Author, Year • Country	% increase in price of	Substitutes: (Demand increases)	Complements: (Demand decreases)	No effect detected
Meta-analysis	• Escobar, 2012 • Global	SSBs	• Fruit Juice: 0.39 (0.19)	• Diet drink: -0.42 (0.15)	• Milk
1	• Grogger, 2015** • Mexico	Sugary soda	• Other drinks with added sugar: 0.63 (0.23)*** • Diet soda: 0.61 (0.13)	• Milk: -0.15 (0.04)***	• Water without added sugar • Pure juice
1	• Ritter Burga, 2016† • Peru	Soda (sugary and diet)		• Food prepared away from home: - 0.07 (0.03)	• Milk • Alcohol • Bottled water and fruit juices
2	• Barquera, 2008†† • Mexico	Sugary soda	• Milk: 0.052 (0.01) •Bottled water: 0.023 (0.00)	• Sweet drinks: -0.122 (0.01) • Juice: -0.016 (0.00)	
2	• Basu, 2014† • India	SSBs (sugary soda, juice)	• Milk: 0.49 (0.02) • Fresh fruit Juice: 0.31 (0.02) • Tea: 0.13 (0.02)		• Coffee
2	• Colchero, 2015† • Mexico	SSBs (sugary soda, juice) + fresh juices	• Milk: 0.19 (0.02) • Natural and mineral water: 0.1 (0.00)	• Candies: -0.44 (0.01) • Snacks: -0.23 (0.01) • Sugar: -0.46 (0.01)	

Estimate (standard error); The following studies are not included in this table: Colchero (2016) because it does not have separate estimates for each non-taxed beverage studied [35], Paraje (2016) because it does not look at substitution effects in a comparable way [44], and Manyema (2012) [17] because it uses meta-analysis estimates for cross-PE from Escobar (2012) [25], presented in the first column

*quasi-experimental studies, coded 1, are considered the highest quality; non-experimental and modeling studies, coded 2, are considered of lower quality

**study estimates changes in price of taxed and untaxed goods relative to 2013 (before the tax was implemented) rather than cross-PEs. The unit of estimates from this study is: change in the product price (in pesos) in 2014 relative to pre-tax the pre-tax price

***Price changes are no longer significant in 2015

†findings are based on an observed increase in the prices of carbonated soft drinks and the unit for estimates presented is liters per month per person given a 10% increase in SSB prices

††estimate presented is a cross-PE for the good listed in the third column, relative to SSBs.

Results for juice were mixed. A modeling study found “fresh fruit juice” to be a substitute to SSBs [40], while “sweet drinks” and “juice” were estimated as complements to sugary soda in a non-experimental study [36]. A quasi-experimental study did not detect relationships between the consumption of sugary soda price and “pure juice” [41].

Bottled water and tea were estimated to be substitutes in two non-experimental studies and one modeling study. Two quasi-experimental studies did not detect a relationship with water [34, 41]; in one study this is likely because fruit juice and bottled water could not be disaggregated [34]. Other foods studied in relation to the prices of SSB products include food prepared away from home, candies, snacks, and sugar [34, 45]. These products were consistently estimated in a quasi-experimental and non-experimental study as complements, suggesting that the full impact on excess energy intake was larger than expected when considering reductions from SSB products alone.

### Objective 3: Measuring Impact on Public Health Outcomes

One quasi-experimental and two modeling studies in this review linked price change to health outcomes to investigate whether market- or policy-based price changes have or will impact obesity outcomes (Table 5). The three studies all showed a negative relationship between price and disease burden. The quasi-experimental study found that, given a six-month average 10% decrease in soda price between 1997 and 2001, obesity prevalence increased by 0.87 percentage points (8.5%) and BMI by 0.12 (14.8%) among adult women between 1997–2001 [34]. One modeling study found that the prevalence of obesity and overweight decreases by about 3% given a 20% tax fully passed on to consumers over 10 years [40]. Another modeling study found that the prevalence of obesity decreases by about 3% given a 20% tax fully passed on to consumers [17].

**Table 5:** Change in Obesity and Overweight Outcomes Given Changes in the Price of SSBs.

Study Type*	Author, Year; Country	Product	Change in price	• Outcome • Unit	Population	Effect	Confidence Level
1	Ritter Burga, 2016;** Peru	Soda	10% average **decrease** in price over 6 months	• Obesity prevalence • Percentage point change	Women 19–49 years old	0.87 (4.76)	p < 0.10
1	Ritter Burga, 2016;** Peru	Soda	10% average **decrease** in price over 6 months	• BMI • Percentage change	Women 19–49 years old	0.12 (0.07)	p < 0.10
2	Basu, 2014; India***	SSBs	20% price **increase,** 2014–2023	• Overweight and obesity prevalence • Percentage change	Full population	-3 (1.1)	p < 0.05
2	Manyema, 2014; South Africa	SSBs	20% price **increase**	• Obesity prevalence • Percentage change	Full population	-3.1 (2.9)	p < 0.05

(Standard Error)

*Quasi-experimental studies, coded 1, are considered the highest quality; non-experimental studies, coded 2, are considered of lower quality

**findings based on observed increase in the prices of soda

***impact modeled over a 10 year period (2014–2023).

## Discussion

This review identified nine studies, including seven studies which had not been included in a previous systematic review. This review fills a gap in the literature on the effectiveness of taxing SSBs by synthesizing results from MICs, where SSB taxes are under strong policy consideration. The key findings from this review are presented in Table 6, and policy recommendations based on these findings are presented in Table 7.

**Table 6:** Summary of Key Findings.

Objective 1: Do the prices of SSBs increase after a tax is imposed?
• Prices of sugary soda increased by more than the rate of the imposed tax in Mexico, ensuring a change in the relative prices faced by consumers. Price increases of sugary fruit drinks were smaller and not sustained. These patterns generally align with other studies from Mexico, Denmark, France, and the United States (U.S.).
Objective 2: How do consumers in MICs respond to higher SSB prices?
• Own-PE estimates for SSB products ranged from -0.6 to -1.2 (median of -0.95). Because this range is slightly lower than a global meta-analysis and similar to a pooled estimate from the U.S., it does not definitively show whether populations in MICs are more or less responsive to price changes of SSBs relative to populations in high-income countries. It does indicate that any differences are likely not large. • Given a 10% increase in price, studies estimated a decrease in SSB consumption equivalent to 5–39 kJ PPPD. • Lower socio-economic groups were more responsive to price changes in SSB products compared to higher socio-economic groups in MICs. • Milk was a likely substitute to SSBs, and foods prepared away from home, snacks, and candy were likely complements. The relationship between the prices of SSB products and various juices and alcohol could not be determined from these studies.
Objective 3: How do SSB prices changes affect population-wide health outcomes in MICs?
• Of the three studies that estimate the effect of changing price on the prevalence of obesity or obesity and overweight, two found a significant change at the 95% confidence level and one at the 90% conference level. While the studies come to relatively consistent conclusions, two of them were modeling studies, and relied on many assumptions, while the quasi-experimental study was based on an observed price decrease which may not have the same magnitude of effect as a price increase.
Overall Quality Assessment
• The number and quality of studies from MICs were insufficient to make definitive conclusions about the effectiveness of the tax. • Studies focused on varying products and outcome measures, often due to limitations in the survey data.

**Table 7:** Policy Recommendations Based on Key Findings.

1.	The increase in SSB prices required to halt the increase in the prevalence of overweight and obesity varies across MICs, with most requiring at least a 20% increase. For permanent change, the tax needs to be implemented in coordination with other obesity prevention interventions.
2.	More empirical research and monitoring of the industry and its response to health-related taxes on SSBs are needed to help policymakers ensure that the increases in consumer prices (and not just the tax rate) are sufficient to reduce population obesity outcomes.
3.	MIC policymakers may consider designing and monitoring a tax on SSBs that avoids exclusive focus on obesity indicators, but instead considers nutritional impact more broadly. For example, policymakers could consider including reductions in the prevalence of type-2 diabetes as an outcome of the tax, given its growth over the last few decades in MICs.
4.	To produce more accurate estimates of impact, more evaluations using longitudinal data and quasi-experimental design are needed.
5.	Adjusting existing household data to draw clearer distinctions could create opportunities for stronger research and avoid some of the limitation faced by some studies in this review (e.g., between beverages with and without added sugar).
6.	To ameliorate any potential burden on the poor, MIC governments designing the tax may want to consider allocating some revenue to support multi-sectoral health promotional activities targeting poor and marginalized populations.

The first objective of this review was to assess the relationship between tax and price. One study from Mexico found the prices of sugary soda increased by more than the tax rate, while the effect on prices of other sugary beverages were smaller and not sustained [41]. Other studies, not in the review, show similar results, which demonstrates the consistency of the results from this review with the wider literature [22, 45, 48, 49]. Three studies (from France, Denmark, and Mexico) used quasi-experimental methods to demonstrate full pass-through or over-shifting of the tax to consumers for sodas overall, though not consistently across all brands, retailers, or regions after six months or more [22, 48, 50]. Another experimental study (from Berkeley, U.S.) found that prices rose by only 69% of the tax rate after three months, but authors expect the price increase to continue [49]. In contrast, juices and other non-carbonated SSB products showed more varied and unstable price patterns after taxes were imposed [22, 49, 50].

However, the market for SSB products in Mexico and the high-income countries studied was relatively consolidated [41, 48, 51, 52]. While to date a small number of multi-national companies dominate the global SSB product market, smaller, regional companies can gain market share [53]. In Peru, a family-run business expanded in the late 1990s, disrupting the market and causing prices for carbonated soft drinks to fall [34]. In India, news agencies report that regional companies control 5% of the market, and in some places have caused multi-national soft drink companies to reduce prices [54]. These trends indicate that the global market for SSBs may be becoming more competitive.

While increases equal to or greater than the tax rate may only be possible in non-competitive markets [21], price increases of less than the tax rate can also reduce SSB consumption. Also, theoretically, SSB prices will remain higher in the long-term even when some new actors enter the market [21]. More empirical research and monitoring of the industry and its response to health-related taxes on SSBs are needed to help policymakers ensure that the increases in consumer prices (and not just the tax rate) are sufficient to reduce population obesity outcomes. This review was limited by the study protocol which may have excluded studies from MICs on this topic.

The second objective of this review was to estimate the change in net intake of energy. Own-PE estimates for SSB products ranged from -0.6 to -1.2, a range slightly lower than a global meta-analysis of SSBs (95% CI: -1.1 to -1.3) [25] and largely overlapped with the pooled mean for soft drinks in the U.S. (95% CI: -0.33 to -1.24) [47]. These comparisons showed no indication that own-PEs for SSB products are higher in MICs than in high-income countries.

This review was not able to definitively determine the effect of complements and substitutes to SSB products on total average reduction of excess calories, given some evidence of substitution to milk on the one hand, and complementary reduction in consumption of food prepared away from home, candies, and snacks on the other. Similar to the findings in this review, a global systematic review indicated milk to be a substitute [23], and a simulation study from the U.S. found that salty snacks and ice cream are complements to SSBs, with the decrease in their consumption accounting for about half of the effect of a 20% increase in SSB prices [55].

A substantial increase in milk consumption may be a positive result in MICs, even if it does not allow for reductions in obesity prevalence. One review argued that SSBs may cause as much harm as they do to health in part through their displacement of milk as a beverage of choice for children, thus reducing the amount of protein and essential vitamins and minerals they consume [3]. Another review argued that children in low-income countries consume insufficient amounts of milk, and that increasing consumption could help address micronutrient deficiencies and promote height and weight gain [56]. In 2014, lower MICs faced similar average levels of stunting among children under five as low-income countries, while also seeing a doubling in the number of overweight children [57]. In this context, MIC policymakers may consider designing and monitoring a tax on SSBs that avoids exclusive focus on obesity indicators, but instead considers nutritional impact more broadly. For example, policymakers may consider reductions in the prevalence of type-2 diabetes as an outcome of the tax, given its growth over the last few decades in MICs [58], as done in the modeling study from India [40] and others from South Africa [59].

Studies quantifying the effect of excess energy intake on bodyweight can help evaluate whether increased SSB prices can impact population health outcomes. Hall et al. (2011) use U.S. data to estimate that the net reduction in excess consumption for adults must be at least 30 kJ PPPD to prevent further increases in obesity prevalence, and at least 100 kJ PPPD for permanent weight reduction, achieved over three years [60]. Applying the 30 kJ benchmark to our results indicates that a 10% increase in price, leading to a 5–39 kJ PPPD decrease in SSB consumption, would be insufficient to prevent further increases in obesity prevalence in MICs, with potential exceptions in Latin American countries such as Ecuador and Mexico.

Comparison of our results with the 100 kJ PPPD benchmark indicate that the tax on SSBs in MICs could only contribute meaningfully to permanent weight loss in a population when implemented alongside other interventions. This understanding of the tax’s potential effectiveness aligns with a 2011 cost-effectiveness study of traffic-light nutrition labeling and a tax on unhealthy foods on obesity prevention from Australia [61]. In this study, Sacks et al. demonstrate how small decreases in consumption across many food groups, including SSBs, can ultimately lead to an effective intervention.

Using findings from Hall et al. (2011) as a benchmark had its limitations. First, the assumption of a linear relationship between reduction in consumption and price increase is unlikely to hold; unfortunately, there is insufficient data available from MICs to address this limitation. In addition, obesity and overweight rates are higher in the U.S. compared to the countries represented in this review [46], which may suggest that some MICs need a larger change in excess energy intake to reduce the prevalence of obesity.

Studies in this review may overestimate the effects of an SSB tax on net energy intake and obesity outcomes. The study from India and the other non-experimental studies calculate PEs based on one year of cross-sectional data and then assumed they were constant over time. However, some studies indicated that such “static” estimation leads to overestimation of own-PE and underestimation of cross-PEs [62]. Other reviews indicated that longitudinal studies are better suited than repeated cross-sectional studies or modeling studies at understanding the relationship between price and consumption [63]. Longitudinal studies are also more capable of detecting small effects. Unlike a longitudinal study from the U.S. [64], the quasi-experimental study based on cross-sectional data in this review was unable to detect a relationship between SSB prices and alcohol consumption [34]. Longitudinal studies may also be more practical than randomized control trials [65]. Also, only three observational studies in this review are quasi-experimental in design; one of them [34] is based on a price drop, and requires the assumption that a price increase will affect body weight to the same degree as a price decrease, which may or may not be true. To produce more accurate estimates of impact and potential unintended consequences like substitution to alcohol, more evaluations using longitudinal data and quasi-experimental designs are needed.

Studies in this review were also limited by overlapping categories of beverages within the datasets they used. For example, potential substitution to juices was not possible to assess in this review because the targeted products included sugary juices, and sometimes non-sugary juices, in only some studies. Similarly, diet soda also had mixed categorization across studies. Studies also varied in the level of clarity they provided on exactly what constituted their categories of “juice.” Adjusting existing household data to draw clearer distinctions, for example, between beverages with and without added sugar could create opportunities for stronger research and avoid some of the limitation faced in this review [34, 45].

In summary, the findings from this review do not resolve the debate among scholars about the nutritional significance of a tax on SSBs. They do indicate at the very least that the tax could help to prevent continued growth of the obesity epidemic, if the tax rate is set sufficiently high. This is true even in a country like India where baseline prevalence of overweight and obesity and sale of SSB per capita per day is low compared to other MICs. These findings suggest that such baseline variables may be important but not definitive in determining the potential effectiveness of an SSB tax on obesity outcomes. The results further indicate that, for permanent change, the tax needs to be implemented in coordination with other obesity prevention interventions.

The review found some indication that lower socio-economic groups or more marginalized populations are relatively more responsive to price changes in SSB products compared to other groups in MICs. These results could indicate that the poorer households who continue to purchase SSBs will spend a larger percentage of their income on the tax than wealthier populations [33]. More research is needed to determine whether an excise tax on SSB will be regressive for poor populations in MICs. To ameliorate any potential burden on the poor, MIC governments designing the tax may want to consider allocating some revenue to support multi-sectoral health promotional activities targeting poor and marginalized populations.

## Conclusions

This review assessed nine studies, including three quasi-experimental studies, and four non-experimental, and two modeling studies. While these studies alone were insufficient to draw strong conclusions about the effectiveness of an SSB tax in improving obesity outcomes in MICs, the review indicates that a tax on SSBs may be a promising policy for MICs to consider in the face of the growing burden of overweight and obesity. A tax on SSBs will increase prices of soda, if not other SSBs, where markets are consolidated. The price increase required to prevent further growth in the prevalence of obesity and overweight varies across MICs, with most needing at least a 20% increase. The review also suggests that the tax alone will not likely lead to reductions in energy intake that are large enough to affect permanent reductions in population weight. The evidence base remains insufficient to definitively determine whether taxing SSBs will be more or less effective in MICs versus high-income countries, though it does indicate that differences will unlikely be large. MIC governments may want to consider adjusting existing surveys to create opportunities for stronger research and evaluation. They may also want to consider including type-2 diabetes as an outcome so that the indicators reflect a holistic understanding of the nutritional needs in MIC populations. They may also consider implementing the tax with other obesity prevention interventions.

## Supporting Information

S1 Fig Own-PEs by Socio-economic Group or Level of Marginality. *Estimates come from the following studies listed as: author, (year of study), country in (year of estimate): 1—Barquera (2008) Mexico in 2006 [36]; 2 –Basu (2014) India 2014–2023 [40]; 3—Claro (2012) in 2003 [39]; 4—Colchero (2015) Mexico across 2006, 2008, and 2010 [42]; 5—Paraje (2016) Ecuador in 2012 [44]; **estimates of kilocalories PPPD given a 10% increase in price for each sub-group were converted to elasticities for this figure; ***standard errors were requested but not received from author; sub-population own-PE estimates in Colchero (2016) are in units that are not comparable with the above estimates. The other studies in this review did not conduct sub-population analysis by socioeconomic group.
(TIF)

S1 Table PRISMA Checklist. (DOCX)
