# Supplementary figures and images for: Taxes on Sugar-Sweetened Beverages to Reduce Overweight and Obesity in Middle-Income Countries: A Systematic Review
Source: PLoS One. 2016 Sep 26;11(9):e0163358. doi: 10.1371/journal.pone.0163358 (PMC5036809; doi:10.1371/journal.pone.0163358)

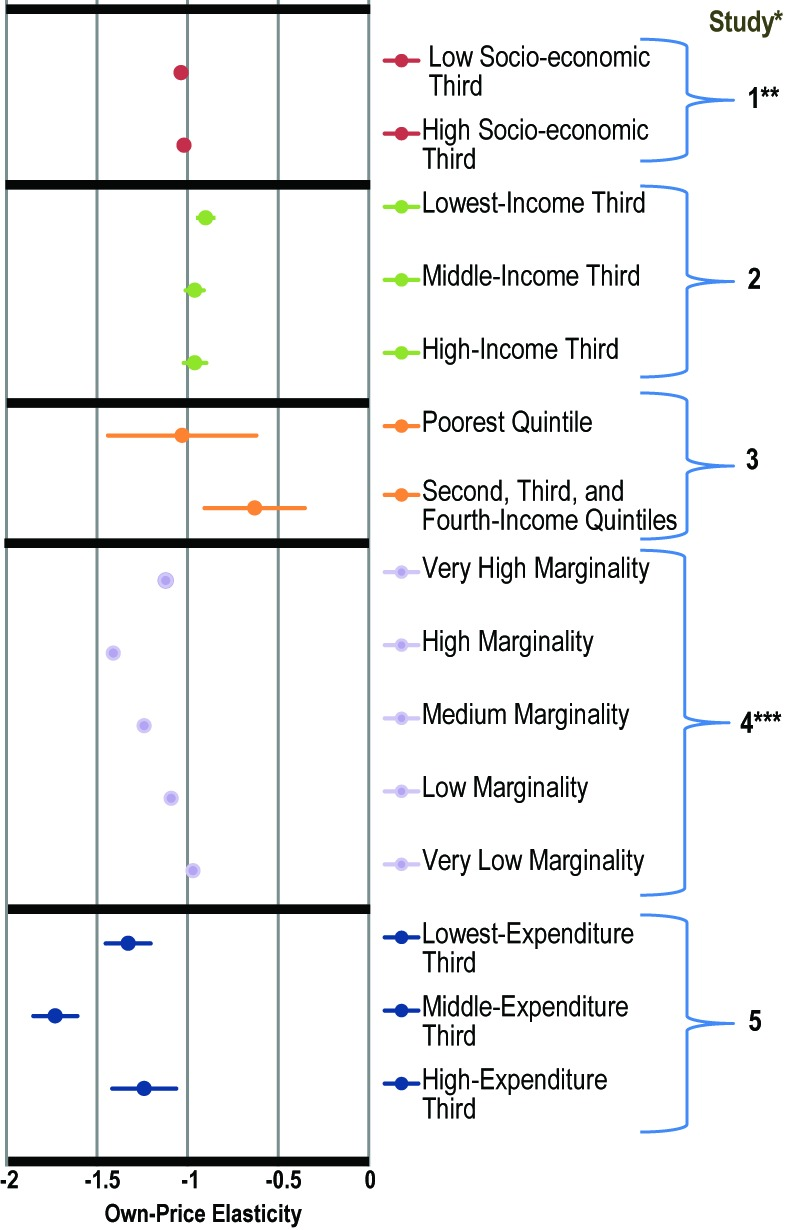

Supplement: S1 Fig — *Estimates come from the following studies listed as: author, (year of study), country in (year of estimate): 1—Barquera (2008) Mexico in 2006 [36]; 2 –Basu (2014) India 2014–2023 [40]; 3—Claro (2012) in 2003 [39]; 4—Colchero (2015) Mexico across 2006, 2008, and 2010 [42]; 5—Paraje (2016) Ecuador in 2012 [44]; **estimates of kilocalories PPPD given a 10% increase in price for each sub-group were converted to elasticities for this figure; ***standard errors were requested but not received from author; sub-population own-PE estimates in Colchero (2016) are in units that are not comparable with the above estimates. The other studies in this review did not conduct sub-population analysis by socioeconomic group. (TIF) [file pone.0163358.s001.tif]
